# Transcriptomic Analysis of the Salivary Glands of an Invasive Whitefly

**DOI:** 10.1371/journal.pone.0039303

**Published:** 2012-06-20

**Authors:** Yun-Lin Su, Jun-Min Li, Meng Li, Jun-Bo Luan, Xiao-Dong Ye, Xiao-Wei Wang, Shu-Sheng Liu

**Affiliations:** 1 Ministry of Agriculture Key Laboratory of Agricultural Entomology, Institute of Insect Sciences, Zhejiang University, Hangzhou, China; 2 Institute of Virology and Biotechnology, Zhejiang Academy of Agricultural Sciences, Hangzhou, China; 3 State Key Laboratory of Identification and Quarantine of Peony Germplasm Resource, Luoyang Entry-Exit Inspection and Quarantine Bureau, Luoyang, China; University of Crete, Greece

## Abstract

**Background:**

Some species of the whitefly *Bemisia tabaci* complex cause tremendous losses to crops worldwide through feeding directly and virus transmission indirectly. The primary salivary glands of whiteflies are critical for their feeding and virus transmission. However, partly due to their tiny size, research on whitefly salivary glands is limited and our knowledge on these glands is scarce.

**Methodology/Principal Findings:**

We sequenced the transcriptome of the primary salivary glands of the Mediterranean species of *B. tabaci* complex using an effective cDNA amplification method in combination with short read sequencing (Illumina). In a single run, we obtained 13,615 unigenes. The quantity of the unigenes obtained from the salivary glands of the whitefly is at least four folds of the salivary gland genes from other plant-sucking insects. To reveal the functions of the primary glands, sequence similarity search and comparisons with the whole transcriptome of the whitefly were performed. The results demonstrated that the genes related to metabolism and transport were significantly enriched in the primary salivary glands. Furthermore, we found that a number of highly expressed genes in the salivary glands might be involved in secretory protein processing, secretion and virus transmission. To identify potential proteins of whitefly saliva, the translated unigenes were put into secretory protein prediction. Finally, 295 genes were predicted to encode secretory proteins and some of them might play important roles in whitefly feeding.

**Conclusions/Significance:**

The combined method of cDNA amplification, Illumina sequencing and *de novo* assembly is suitable for transcriptomic analysis of tiny organs in insects. Through analysis of the transcriptome, genomic features of the primary salivary glands were dissected and biologically important proteins, especially secreted proteins, were predicted. Our findings provide substantial sequence information for the primary salivary glands of whiteflies and will be the basis for future studies on whitefly-plant interactions and virus transmission.

## Introduction

A wide range of hemipterans feed on phloem sap with their highly modified piecing-sucking mouthparts. During the process of feeding, complex interactions between insects and plants are established. Saliva injected into plant tissues through stylets of phloem feeders has long been considered to play crucial roles in aiding penetration, ingesting nutrient and modulating plant responses, and some secreted proteins with structural, enzymatic or chelating features are found as the effectors of these phenomena [1]–[3]. Since most of hemipteran herbivores act as vectors of pathogens, phytopathogens can be also secreted and inoculated into healthy plants through insect salivation [4]. Thus, the saliva of phloem feeders is a mediator of plant- (pathogen-) insect interactions and the salivary glands, the key organs for secretory substance production and delivery, are indispensable in insect feeding and pathogen transmission [4].

The whitefly *Bemisia tabaci* (Gennadius) (Hemiptera: Aleyrodidae) is a genetically diverse species complex with a global distribution [5], [6]. Some species of this complex are important pests of crops [5], [7]–[9]. For example, the Mediterranean cryptic species (herein MED, formerly referred to as the Q ‘biotype’), one of the most remarkable and destructive members of *B. tabaci*, has invaded many parts of the world and has caused great loss in agriculture [10]–[13]. Species of the *B. tabaci* complex impair plants mainly by excessive sap consumption and plant virus transmission [14], [15]. Their successful feeding and extensive damages to plants are likely enabled by whitefly saliva.

Whiteflies, as well as other phloem feeders, secrete two kinds of saliva during the process of feeding: gelling saliva and watery saliva [16]. The gelling saliva is released during stylet insertion, and forms sheath enclosing stylet bundles, whereas the watery saliva is secreted after penetration of a sieve element [16]. Both kinds of saliva are speculated to be important in whitefly-plant interactions. Nevertheless, due to the difficulties of saliva collection, only the activity of alkaline phosphatases has been detected in the saliva of the whitefly Middle East-Asia Minor 1 (herein MEAM1, formerly referred to as the B ‘biotype’), but the role of this enzymes is still unidentified [17]. The organs of whiteflies for saliva generation are a pair of primary and accessory salivary glands [18]. The primary salivary glands are compact and kidney shaped [19], [20]. The types of cells in these glands are complex and most of the cells have been supposed to have high levels of metabolic activity and be involved in synthesis of secretory macromolecules, and thus the primary salivary glands are believed to be the main sites for production and secretion of salivary components [20]. Except for the role of salivation, previous studies on virus transmission in whiteflies indicated that the primary salivary glands, but not the accessory salivary glands, are the crucial organs for harboring and transmission of virions [19], [21]–[23]. Although the whitefly primary salivary glands have been carefully characterized at the anatomical level, the biological features and molecular constituents of these organs are almost unknown, let alone the mechanisms of saliva production, secretion and the proteins involved in plant-insect interactions and virus transmission.

Transcriptomic analysis has been applied to study the salivary glands of several hemipterans, and many proteins (including secretory proteins) have been identified. However, the previous studies on the transcriptomes of hemipteran salivary glands generally produced only about 2,000 genes due to the throughput limitation of traditional sequencing approach [24]–[30]. As a result of the low redundancy of sequencing reads, these data can not be used for estimation of gene expression profiles based on transcript abundance [31], [32]. Thus, a more effective and low-cost method is required to promote research in this field. The high-throughput next generation sequencing technology makes it possible to carry out large-scale gene discovery and gene expression profiling studies in an efficient and precise way [33]–[36]. Recently, the feasibility of this technology has been proven in our previous studies on the whitefly transcriptomes [37], [38].

For the next generation sequencing, microgram amounts of total RNA are required, which correspond to hundreds of thousands of cells [39]. A primary salivary gland of whitefly contains only 13–20 cells. It means that nearly ten thousands of primary glands have to be dissected for RNA extraction, and workload of dissection makes the study of whitefly salivary gland transcriptome extremely difficult and inefficient. Coincidently, the development of cDNA amplification technique offers a great opportunity to investigate the transcriptome and gene expression profiles with limited material, even picogram of total RNA [40]. The switching mechanism at 5′ end of RNA template (SMART) PCR amplification has been commonly used for cDNA amplification. This technique is fast, cost effective with unlimited degree of amplification and increased length of cDNA [41], [42]. Although SMART cDNA amplification has been successfully applied to characterize the transcriptomes of cultured cells using Illumina sequencing platform [43], this technique has never been used for large-scale gene discovery in an organism without a reference genome.

Here, a combination of SMART PCR amplification and Illumina sequencing was utilized to generate the transcriptome of whitefly primary salivary glands. In a single run, we produced almost 13 million of high quality DNA reads for the primary salivary glands of the MED whitefly. After *de novo* assembly and annotation, 13,615 unigenes were produced, of which 3,159 were annotatable against the non-redundant (nr) NCBI nucleotide database (E-value<1.0E^−5^). Through the analysis of the transcriptome data, genomic features of the primary salivary glands were dissected and the genes encoding biologically important proteins, especially secreted proteins, were identified. This study provides a rich molecular resource for future functional studies on primary salivary glands and will contribute to a better understanding of whitefly-plant interactions and virus transmission.

## Results and Discussion

### Illumina Sequencing and Reads Assembly

To analyze the transcriptome of whitefly salivary glands, the cDNA sample of the MED whitefly primary salivary glands was sequenced using the Illumina sequencing platform. Through filtering the adaptors and low quality sequences, about 13 million of 90 bp reads were obtained. These reads were subsequently assembled using SOAPdenovo software and resulted in 22,239 scaffolds (Table 1). After gap-filling and clustering, finally 13,615 unigenes (unique sequences obtained after assembly of all the reads) (Table 1) were generated from these scaffolds with the size ranging from 150 bp to 2,345 bp (Figure S1). The total number of unigenes obtained from the salivary glands of the whiteflies is at least four folds that of other plant sap-sucking insects, which may be attributable to the advantages of the next generation sequencing [27], [30], [44].

**Table 1:** Summary for the MED whitefly primary salivary gland transcriptome.

Total number of reads	12,944,446
Total base pairs (bp)	1,165,000,140
Average read length (bp)	90
Total number of contigs	199,403
Total number of scaffolds	22,239
Mean length of scaffolds (bp)	229
Total unique sequences	13,615
Mean length of unique sequences (bp)	297

### Annotation of Primary Salivary Gland Transcripts

**Figure 1:**
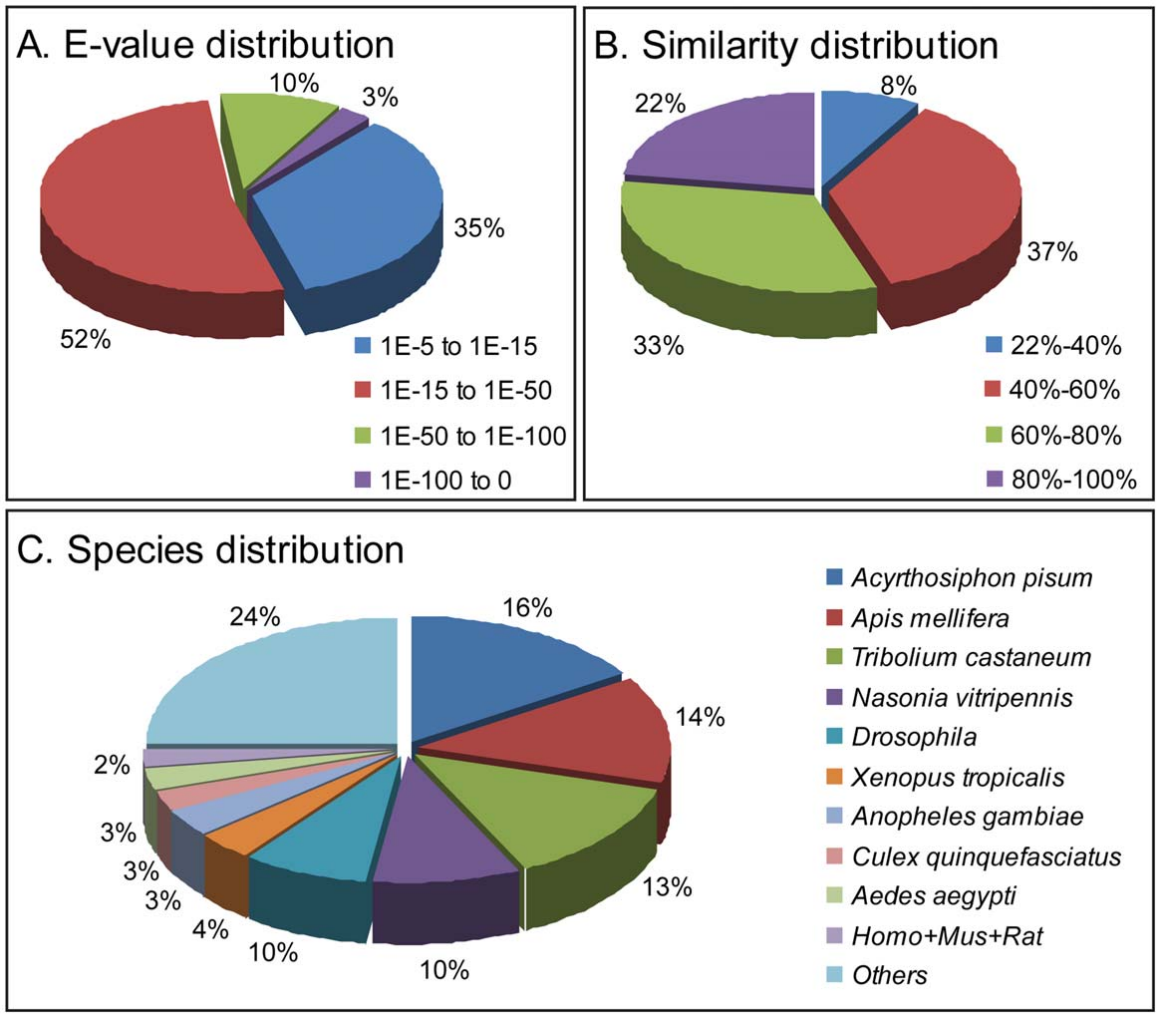
Characteristics of homology search of Illumina sequences against the nr database. (A) E-value distribution of BLAST hits for each unique sequence with a cut-off E-value of 1.0E^−5^. (B) Similarity distribution of the top BLAST hits for each sequence. (C) Species distribution is shown as a percentage of the total homologous sequences with an E-value of at least 1.0E^−5^. We used the first hit of each sequence for analysis. Homo: *Homo sapiens*; Mus: *Mus musculus*; Rat: *Rattus norvegicus*.

For functional annotation, the 13,615 unigenes were searched using BLASTx against the nr NCBI nucleotide database and a total of 3,159 unigenes returned significant BLAST hits (E-value<1.0E^−5^) (Table S1). The E-value distribution of the best hits against the nr database showed that 13% of the sequences have strong homology (E-value<1.0E^−50^), and the E-values of most of the sequences range from 1.0E^−15^ to 1.0E^−50^ (Figure 1A). On the other hand, the similarity distribution demonstrated that 55% of the unique sequences with best hits have a similarity higher than 60%, while 45% of the hits have a similarity ranging from 22% to 60% (Figure 1B). For the species distribution, we found that the highest percentage of the primary gland unique sequences are matched to the genes of the pea aphid *Acyrthosiphon pisum* (16%), followed by the honey bee *Apis mellifera* (14%), the red flour beetle *Tibolium castaneum* (13%) and the wasp *Nasonia vitripennis* (10%) (Figure 1C).

### Assignment of Unigenes to Gene Ontology (GO) Terms and Kyoto Encyclopedia of Genes and Genomes (KEGG) Pathways

**Figure 2:**
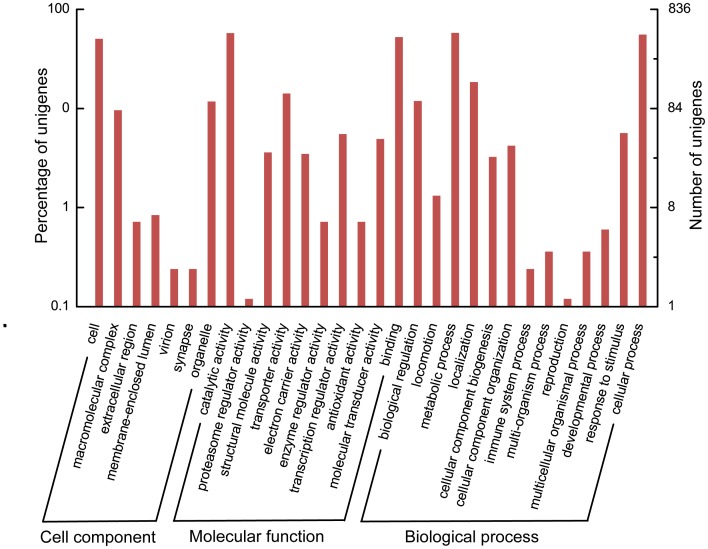
Histogram presentation of GO classification of genes from the primary salivary glands. The results are summarized in three main categories: biological process, cellular component and molecular function. The right y-axis indicates the number of genes in a category. The left y-axis indicates the percentage of a specific category of genes in that main category.

**Figure 3:**
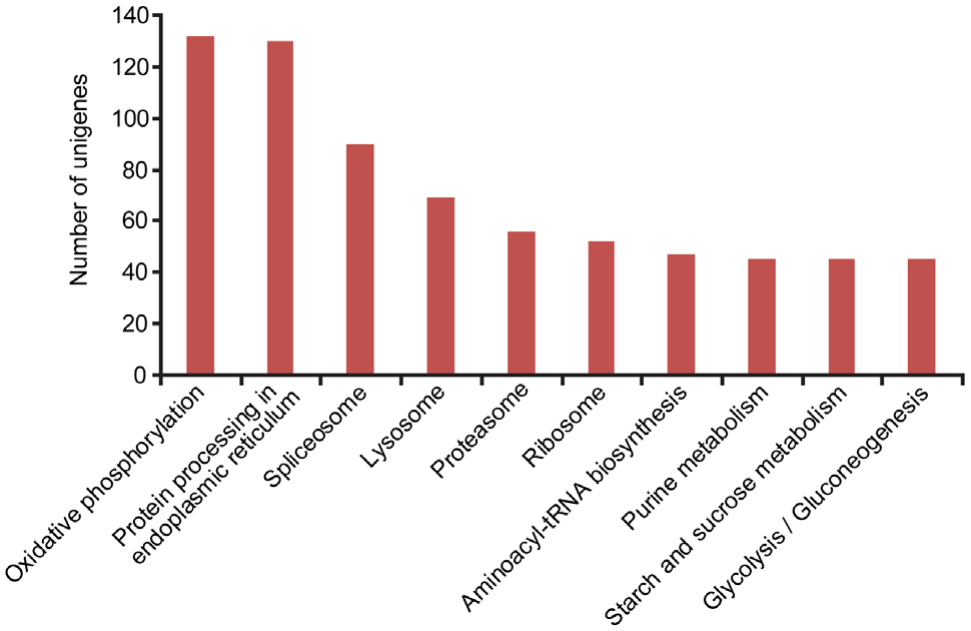
Distribution of unigenes from the primary salivary glands among the KEGG pathways. The top ten pathways (excluding disease related) with highest percentages of unigenes mapped to are shown.

GO assignments were used for the functional classification of the predicted proteins. Based on sequence homology, 836 sequences with GO annotations were categorized into 30 groups at level two. The three most basic ‘Cell component’ categories are ‘Cell’, ‘Organelle’ and ‘Macromolecular complex’. In ‘Molecular function’, the three most common categories are ‘Catalytic activity’, ‘Binding’ and ‘Transporter activity’, while in ‘Biological process’, the top three are ‘Metabolic process’, ‘Cellular process’ and ‘Localization’, respectively (Figure 2). To investigate which biological pathways are active in the primary salivary glands, the 13,615 unigenes were assigned to the reference canonical pathways in KEGG. Consequently, 2,047 unigenes were mapped to 201 pathways in total. Among these pathways, ‘Oxidative phosphorylation’ includes the highest percentage of unigenes (130 unigenes), followed by ‘Protein processing in endoplasmic reticulum’ (115 unigenes) and ‘Spliceosome’ (90 unigenes) (Figure 3, human diseases associated pathways were excluded). The results of GO annotations and KEGG mapping indicate that the primary salivary glands might be active in metabolism and transport.

### Statistical Analysis of Enriched Ontologies and Pathways

In order to gain an insight into the physiological characters of the primary salivary glands, a hypergeometric test was performed to explore statistically enriched GO terms in the primary salivary gland unigenes compared to the whole-body transcriptome background [45]. The results are shown in Table 2, Table S2 and Table S3 (*p*-value≤5.0E^−3^). In the category of ‘Molecular Function’, proteins with transporter activity were highly enriched in the primary salivary glands at level two, followed by ‘Transcription regulator activity’, ‘Catalytic activity’ and ‘Electron carrier activity’ (Table 2). In the ‘Transporter activity’ category, nine ontologies were found enriched including ‘Secondary active transmembrane transporter activity’, ‘Active transmembrane transporter activity’, ‘Symporter activity’ and ‘Solute: cation symporter activity’ (Table 2). From the category of ‘Biological Process’, 58 ontologies were found enriched and 47% of them belong to metabolism followed by biological regulation (14 ontologies) and location (11 ontologies) (Table S2). In addition, the analysis of multilevel ‘Cell Component’ enrichment indicated that plasma membrane is the prominent part of the primary salivary glands (Table S3).

**Table 2:** Statistically enriched Gene Ontology terms in the “Molecular function=" category.

GO ID	SG Genes^1^	WB Genes^2^	*p*-value	GO Ontology or Category
0005215	118	240	1.96E-10	Transporter activity
0022804	44	69	8.88E-09	Active transmembrane transporter activity
0022884	8	8	7.60E-05	Macromolecule transmembrane transporter activity
0015399	25	41	5.02E-05	Primary active transmembrane transporter activity
0015405	25	41	5.02E-05	P-P-bond-hydrolysis-driven transmembrane transporter activity
0008320	8	8	7.60E-05	Protein transmembrane transporter activity
0015450	8	8	7.60E-05	P-P-bond-hydrolysis-driven protein transmembrane transporter activity
0015291	19	20	2.13E-09	Secondary active transmembrane transporter activity
0015293	14	16	3.68E-06	Symporter activity
0015294	12	14	3.06E-05	Solute: cation symporter activity
0030528	46	72	3.66E-09	Transcription regulator activity
0003824	479	1381	5.28E-07	Catalytic activity
0016740	151	320	1.91E-11	Transferase activity
0016746	22	27	6.05E-08	Transferase activity, transferring acyl groups
0016747	18	21	2.41E-07	Transferase activity, transferring acyl groups other than amino-acyl groups
0016407	7	9	4.84E-03	Acetyltransferase activity
0016765	8	10	1.82E-03	Transferase activity, transferring alkyl or aryl (other than methyl) groups
0016741	28	32	2.71E-11	Transferase activity, transferring one-carbon groups
0008168	28	28	2.89E-15	Methyltransferase activity
0016779	22	39	6.76E-04	Nucleotidyltransferase activity
0034061	7	8	1.46E-03	DNA polymerase activity
0016853	30	52	4.02E-05	Isomerase activity
0016866	10	14	1.94E-03	Intramolecular transferase activity
0016491	98	255	3.15E-03	Oxidoreductase activity
0042623	28	58	3.31E-03	ATPase activity, coupled
0003678	6	7	4.23E-03	DNA helicase activity
0009055	29	54	3.06E-04	Electron carrier activity
0005488				Binding
0008289	12	13	6.06E-06	Lipid Binding
0051082	13	21	2.90E-03	Unfolded protein binding
0050662	27	55	2.89E-03	Coenzyme binding
0003676	150	388	1.53E-04	Nucleic acid binding
0003677	94	176	5.75E-11	DNA binding
0051540	16	26	1.04E-03	Metal cluster binding
0051536	16	26	1.04E-03	Iron-sulfur cluster binding
0051539	9	11	6.72E-04	4 iron, 4 sulfur cluster binding
0000287	18	26	5.30E-05	Magnesium ion binding
				Others
0003700	36	48	2.12E-10	Transcription factor activity

1 The number of primary salivary gland (SG) genes that belong to each GO.

2 The number of whole-body (WB) genes that belong to each GO.

Enrichment analysis is also an effective tool to identify the pathways that frequently occur in the set of salivary gland genes mapped to KEGG orthologs with the whole body transcript distribution as background [45], [46]. Totally, 27 salivary gland enriched pathways (*p*-value<5.0E^−3^) were identified (Table 3). At level two, ‘Transcription’, ‘Translation’, ‘Energy Metabolism’, ‘Folding, Sorting and Degradation’, ‘Transport and Catabolism’ and ‘Environmental adaptation’ are the enriched pathways in the primary salivary glands (Table 3). Among them, ‘Translation’, ‘Folding, Sorting and Degradation’ and ‘Energy Metabolism’ have the lowest *p*-values (Table 3).

**Table 3:** Statistically enriched KEGG pathways.

KEGG Pathway	*p*-value	SGGenes^1^	WBGenes^2^
Genetic Information Processing	0.00E+00	979	3747
Transcription	2.43E−03	217	1160
Translation	0.00E+00	254	861
RNA transport	0.00E+00	97	134
mRNA surveillance pathway	0.00E+00	31	32
Aminoacyl-tRNA biosynthesis	5.18E−07	47	146
Ribosome biogenesis in eukaryotes	7.93E−05	13	27
Folding, Sorting and Degradation	0.00E+00	456	1281
RNA degradation	0.00E+00	46	70
Protein processing in endoplasmicreticulum	0.00E+00	113	242
Proteasome	0.00E+00	56	63
Protein export	0.00E+00	34	42
SNARE interactions in vesicular transport	1.01E−04	14	31
Cellular Processes			
Transport and Catabolism	1.32E−09	147	585
Lysosome	4.53E−10	59	168
Peroxisome	1.60E−06	27	68
Phagosome	7.12E−04	42	164
Metabolism			
Energy Metabolism	0.00E+00	176	389
Environmental Adaptation	7.15E−08	32	78
Others			
Lysine degradation	3.77E−08	30	69
Tryptophan metabolism	7.76E−06	26	69
Valine, leucine and isoleucinedegradation	1.96E−03	27	98
beta-Alanine metabolism	1.34E−03	18	55
Glycolysis/Gluconeogenesis	3.51E−09	43	111
Citrate cycle (TCA cycle)	1.40E−08	34	81
Pyruvate metabolism	2.50E−03	25	90
Fatty acid metabolism	6.54E−07	29	73
Fatty acid elongation in mitochondria	3.28E−07	11	14

1 The number of salivary gland (SG) genes that belong to each KEGG pathway.

2 The number of whole-body (WB) genes that belong to each KEGG pathway.

Saliva secretion is the main function of the primary salivary glands. Coherently, a large number of enriched ontologies and pathways we found are correlative with transport and secretion (Table 2, 3 and Table S2, S3). Hemipteran saliva is composed of water, electrolytes, lipids, amino acids and proteins [2]. For fluid and electrolyte secretion, many transporters are involved, such as the primary active transporters: V-ATPases and Na^+^/K^+^-ATPases and the secondary active transporters: Ca^2+^-ATPases and Na^+^/K^+^/Cl^–^co-transporters [47], [48]. As a result, enrichments of transporters are necessary for the regulation of secretion. Besides fluid and electrolyte secretion, macromolecule secretion is also critical. In insect salivary glands, clusters of secretory granules and vesicles containing proteins and lipids are accumulated to the apical microvilli of salivary glands and secreted rapidly through budding vesicles when they are needed [20], [49], [50]. Here, we found that ‘Plasma membrane’, ‘SNARE interactions in vesicular transport’ and eight ontologies of protein transport are enriched and might be closely related to intracellular trafficking of secretory vesicles and exocytosis (Tables S2 and S3, and Table 3) [48], [51]. As the primary salivary glands are crucial sites for virus harboring, the proteins involved in these enriched groups might be exploited by plant viruses to facilitate their transmission. Future studies on the roles of these proteins during virus transmission may contribute to the development of novel approaches for plant virus control.

The primary salivary glands are specific organs for salivary macromolecule production and consequently have a high level of metabolic activity. This character was implied in a previous study through the observation of the dense cytoplasm and organized whorls of rough endoplasmic reticulum in the salivary gland cells [20]. Coincidently, enrichment analysis of the GO terms and the KEGG pathways in biological process suggests that metabolism and the regulation of metabolism are predominantly important in the primary salivary glands (Table 3, Table S2). Since there are abundant proteins in the saliva of whiteflies, as one of the origin of these proteins, the primary salivary glands should be active in protein synthesis and catabolism. Indeed, many groups of the genes involved in protein metabolism were enriched in the primary salivary glands. For example, ‘Protein processing in endoplasmic reticulum’ and ‘Protein folding’ are related to the folding of nascent secretory proteins (Table 3, Table S2). ‘Proteasome’ and ‘Lysosome’ (Table 3) are the two major paths for short-lived and long-lived protein degradation [52]–[54], which are essential to cells for maintaining homeostasis of the primary salivary glands when new proteins are synthesized.

### Unigenes Encoding Putative Secreted Proteins

To identify putative secreted proteins, all unique genes were analyzed for the presence of signal peptide and potential cleavage site. Totally, 356 unique genes were found to encode a secretory signal peptide (Table S4), comparable to the number of putative secreted proteins reported in the pea aphid salivary gland transcriptome (324 genes) [30]. Out of these 356 genes, 61 were predicted to comprise at least one transmembrane domain besides the signal peptide and these proteins are likely embedded in cell membranes of the salivary glands. After removing those sequences, 295 potential secretory proteins were retained (Table S4). Interestingly, the possible functions of some putative secreted proteins are closely related to the known roles of insect saliva, such as digestion and eliciting or suppression of plant defense. In the salivary gland transcriptome, we identified a secretory oligo-1,6-glucosidase which has been proved to be secreted in the guts of cockroaches and beetles and may play a part in carbohydrate digestion [55], [56]. In addition, we noticed a serine protease among the putative secreted proteins. Because a variety of proteins has been detected and the concentration of proteins can reach quite high level in plant phloem [57]–[59], a secretory serine protease may function in protein digestion.

Several substances of herbivore saliva, such as dehydrogenases and disulfide-isomerases, may induce plant responses. Dehydrogenases, including NADH dehydrogenases, Zn-dependent alcohol dehydrogenases and short-chain dehydrogenase/reductases (SDRs), found in our transcriptome have also been documented in many studies on aphid saliva [30], [60], [61]. Among them, SDRs were reported to be involved in the biosynthesis of abscisic acid which is important to plant responses under stresses and may affect the release of jasmonic acid precursors [62], [63]. In *Arabidopsis thaliana*, a SDR has been identified to be up-regulated after aphid feeding [64]. Due to the crucial role of SDRs in plants, the presence of these enzymes in the putative secreted proteins of whiteflies raises a possibility that they may have an impact on plant cellular signaling and act as an elicitor of stress response. In the study of interactions between fungi and plants, disulfide-isomerases were speculated to have a chaperone function and to be involved in plant disease-signaling pathways [65]. Secreted disulfide-isomerases were both found in the pea aphid secretome [30] and in the transcriptome of the whitefly salivary glands. Therefore, this kind of enzymes might be able to trigger plant defense as well.

As the consequence of co-evolution, some components should exist in whitefly saliva to prevent plant defenses and aid whiteflies to ingest phloem sap. Sieve tubes are sensitive to injuries. After wounding, abundant calcium influx into the sieve-element lumen and the increase of calcium concentration finally triggers the occlusion of sieve plates [66]–[68]. Aphids can successfully puncture sieve tubes with their stylets and ingest phloem sap without triggering the occlusion of sieve tubes. Previous studies indicated that this was owning to the calcium-binding proteins in aphid saliva, which can inhibit the occlusion of sieve tubes caused by aphid feeding [30], [69], [70]. The functional annotation of whitefly salivary gland unigenes suggests that a predicted secretory protein, soluble calcium-activated nucleotidase 1, contains calcium-binding domains. It is likely that this calcium-binding protein has a function in whitefly feeding similar to that of aphids. Wounds not only trigger direct calcium release in cells, but also induce extracellular ATP accumulation that increases the cytosolic calcium concentration and subsequently elicits plant defense response as well [71]. Several putative secretory proteins with ATP hydrolysis function, such as soluble calcium-activated nucleotidase 1 and 5-nucleotidase, were found in the primary salivary glands of whiteflies. Therefore, these proteins might be implicated in reducing the concentration of extracellular ATP and preventing plant response during whitefly feeding. Detoxification is another way for whiteflies to respond to plant defensive compounds and utilize phloem sap. Sulfatases are the enzymes of the esterase class that hydrolyze sulfate esters of a wide range of substrates, including carbohydrates, steroids and proteins. Interestingly, in order to suppress the defense of cruciferous host plants, *Plutella xylostella* utilizes a sulfatase to modify glucosinolates in plants and then, prevent the formation of toxic products by glucosinolate hydrolysis [72]. Since some whitefly species including the MED whitefly are able to feed and survive on crucifer plants, sulfatases may aid the detoxification of toxin in phloem sap.

Through this analysis, we identified a number of secreted proteins that might have important functions in whitefly-plant interactions. As both whiteflies and aphids are phloem-sucking insects capable of secreting gelling and watery saliva during feeding, these two groups of insects are hypothesized to have some similarities in the proteins of saliva. In order to gain an insight of that hypothesis, we analyzed the secretory proteins of those two groups of insects. In previous studies, great efforts have been made to characterize the proteins of aphid saliva, including bioassays and mass spectrometry of aphid saliva [60], [61], [70], [73], [74], a dual transcriptomic/proteomic approach for aphid secretome study [30], RNA interference in aphids [75], [76], and overexpression of candidate salivary effectors in plants [77]. Various secretory proteins of different aphid species have been identified. Herein, we compared the translated protein sequences of the whitefly primary salivary glands to the sequences of aphid secretory proteins, and found some similar sequences (E-value≤1.0E^−5^) between the two sets of data (Table S5).

Sixty predicted protein sequences of the whitefly primary salivary glands were identified to have a significant BLAST hit to the protein sequences associated with aphid saliva. Most of the translated unigenes were matched to the aphid protein sequences for sucrases/amylases (21 unigenes), followed by proteases (including metalloproteases and angiotensin converting enzymes) (10 unigenes) [70], dehydrogenases (9 unigenes), esterases (7 uingenes) and regucalcins (3 unigenes) (Table S5), all of which have been speculated to be involved in digestion, modification of plant defenses and detoxification [30], [61], [70]. However, homologs of a number of known aphid salivary effectors, such as C002, SHP, Mp10 and Mp42, which have positive or negative impacts on aphid feeding, were not identified in our study [70], [75], [77]. Considering some proteins of saliva are species-specific [30], [75], [77], whiteflies might have developed their own set of salivary effectors which are different from those in aphids during the co-evolution between whiteflies and their plant hosts. In this comparison, although many homologs of aphid secretory proteins were found in our transcriptome, only three of them were annotated to encode secretory proteins (Table S5). A possible reason is that aphids and whiteflies use distinct strategies in feeding, which makes the secretory proteins of the two groups of insects different except for a carbohydrate digestive enzyme and a dehydrogenase. In addition, there exists another possibility that many genes in our transcriptome might encode secretory proteins and these proteins are homologous to the proteins of aphids, but these genes were filtered out during of the prediction of secretory proteins due to their incomplete 5′ sequences. For example, the activity of alkaline phosphatases has been detected in the salivary glands of whiteflies in a previous study [17], and in our data, a unigene (BT_Q_SG_ZJU_Unigene856) was also annotated to encode an alkaline phosphatase, but due to the lacking of 5′ end, it was filtered out during the signal peptide prediction.

### Changes in Gene Expression Profile between Primary Salivary Glands and Whole Body

**Figure 4:**
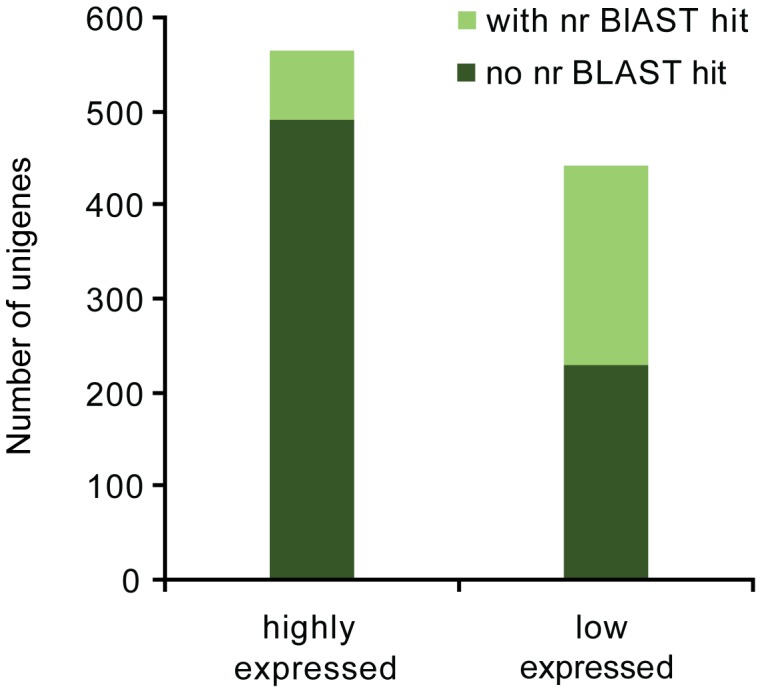
Changes in gene expression profile between the primary salivary glands and the whole body. The histogram summarized the numbers of highly expressed and low expressed genes in the primary salivary glands that hit and did not hit to nr database.

Gene expression levels can be estimated from Illumina sequencing based on the number of raw reads for a gene [78], [79]. In addition, SMART is an amplification method that systematically compresses observed gene expression ratios, but has high true-discovery rate of differentially expressed genes [80]. Therefore, the SMART-amplified cDNA of the whitefly primary salivary glands could be used for identification of differentially expressed genes. To compare the level of gene expression between the primary salivary glands and the whole body, the orthologous gene pairs between the whole body and the salivary gland transcriptomes were identified by MegaBLAST. Then, the number of reads mapped to the orthologous region of each gene pair was summed and normalized using the Reads Per Kilobase per Million mapped reads (RPKM). Using the method described by Chen *et al.* [81], gene expression values were measured, and 1,008 genes were identified to be differentially expressed between the salivary glands and the whole body transcriptomes (FDR<1.0E^−3^) (Table S6). Compared to the whole body transcriptome, 565 genes were highly expressed while 443 were low expressed in the salivary glands. Among them, 48.3% (214 genes) of low expressed unigenes have a significant hit against the nr database and Swissprot database, whereas only 13.1% (74 genes) of highly expressed genes could be annotated (E-value<1.0E^−5^) (Figure 4). Obviously, the percentage of non-annotatable genes of highly expressed genes in the salivary glands is much higher than that of low expressed genes, which may be due to the fact that the highly expressed genes encode tissue-specific effectors in the primary salivary glands. To validate the gene expression data obtained through statistical comparison of RPKM value, we compared the gene expression profiles of the primary salivary glands and the whole body (minus primary salivary glands) using quantitative PCR (qPCR). Out of the 20 selected genes, 19 showed concordant direction of change between the statistical comparison and qPCR results (Table S7).

In order to understand the functions of highly expressed genes, these genes were searched against nr and Swissprot database. Among the 74 sequences with annotation, we identified 15 unigenes that encode proteins closely related to protein secretion (Table 4). Regulation of saliva secretion ensures the release of salivary macromolecules when they are in need during feeding, and the extracellular stimulation transduction is a key step in this process. Salivary gland activities of a hemipteran *Rhodnius prolixus* were considered to be controlled by neural stimulation [82]. In this study, two genes encoding neurotransmitter gated ion channels were expressed about 23 and 37 folds higher respectively compared with their transcripts in the whole body transcriptome (Table 4). Meanwhile, cation-transporting ATPases which might be involved in secretion regulation by supplying the electromotive force were also found at a high expression level in the primary salivary glands [83], [84].

**Table 4:** Highly expressed unigenes related to secretion regulation and secretory protein processing and trafficking.

Gene ID^1^	Species	Accession No.	Annotation	FC^2^
Unigene12942	*Bombyx mori*	NP_001040188.1	KDEL receptor 2	2.30
Unigene13103	*Saccoglossus kowalevskii*	XP_002737127.1	SIL1 protein-like	2.75
Unigene8460	*Apis mellifera*	XP_623191.1	similar to ERGIC-53	2.79
Unigene12156	*Glyptapanteles indiensis*	ACE75391.1	cation-transporting ATPase	3.03
Unigene1123	*Mus musculus*	Q9CR89	ERGIC-2	4.14
Unigene6526	*Lepeophtheirus salmonis*	ACO12387.1	signal peptidase complex subunit 3	4.20
Unigene13193	*Aedes aegypti*	ABF18277.1	DNAJ chaperone	4.50
Unigene12279	*Acyrthosiphon pisum*	XP_001943684.1	similar to arf6 guanine nucleotide exchange factor	4.63
Unigene12741	*Caligus clemensi*	ACO15053.1	signal peptidase complex subunit 1	6.59
Unigene13392	*Acyrthosiphon pisum*	NP_001119639.1	Sec61 alpha 1 subunit	6.68
Unigene12253	*Aedes aegypti*	XP_001654826.1	dolichyl-diphosphooligosaccharide protein glycosyltransferase	7.06
Unigene11747	*Rattus norvegicus*	O54880	Rab effector Noc2	9.71
Unigene496	*Tribolium castaneum*	XP_969111.2	similar to alpha-(1,6)-fucosyltransferase	9.78
Unigene5624	*Acyrthosiphon pisum*	XP_001950325.1	similar to neurotransmitter gated ion channel	23.26
Unigene9390	*Acyrthosiphon pisum*	XP_001950325.1	similar to neurotransmitter gated ion channel	36.76

1 Unigene: BT_Q_SG_ZJU_Unigene.

2 FC: fold change of gene expression.

For secretion, proteins involved in particle trafficking in the secretory pathway are supposed to be highly expressed. Consistent with this, we found the Sec61 alpha 1, ERGIC-53, ERGIC-2, KDEL receptor 2 which facilitate protein insertion to the endoplasmic reticulum (ER) and ER-Golgi transport were highly expressed in the primary salivary glands compared with the whole body. Another two genes, arf6 guanine nucleotide exchange factor and Rab effector Noc2 that probably are involved in endocytosis and exocytosis were conspicuously up-regulated in the salivary glands as well. Arf6 guanine nucleotide exchange factor is an activator of Arf6 and its high expression (4.63 folds) might suggest that Arf6-mediacted endocytosis is active in the primary glands [85], [86]. In addition, previous research proposed that Arf6 is an important protein for efficient entry and infection of some human viruses [87], [88]. Therefore, the possibility that plant viruses utilize Arf6 guanine nucleotide exchange factor for salivary gland entry can not be excluded. Rab effector Noc2 is a protein with a function of exocytosis regulation [89]. In a mammal study, with the inhibition of Noc2, secretory granules accumulated remarkably and amylase release was significantly impaired [89]–[91].

As most of the proteins of saliva originate from cells of salivary glands, protein processing should be active in the primary glands. To our knowledge, signal peptidase complex has a key function in the cleavage of signal peptides from the nascent exported proteins in the ER. Coincidently, subunits 1 and 3 of the signal peptidase complex were found to be expressed 6.59 and 4.20 folds higher in the primary glands compared with the whole body (Table 4). Meanwhile, DNAJ chaperone, SIL1 protein, dolichyl-diphosphooligosaccharide protein glycosyltransferase and alpha-(1,6)-fucosyltransferase, which are probably correlative with protein folding and modification, were expressed significantly higher in the primary glands than in the whole body. Among these proteins, the SIL1 was also found highly expressed in kidney, liver and placenta of mammals, which produce large amounts of secreted proteins to control protein folding [92]. Such high expression of the genes involved in protein processing, transport and secretion reflects that protein secretion is critical in the salivary glands of whiteflies.

### Conclusion

In this study, we sequenced the transcriptome of the primary salivary glands (an organ with only 13–20 cells) of the MED species of the whitefly *B. tabaci* complex using an effective cDNA amplification method in combination with Illumina sequencing technology. In a single run, we obtained 13,615 unique sequences including 3,159 sequences with significant nr BLAST hits. Consistent with earlier morphological studies, the enrichment analysis highlighted that metabolism and transport occur frequently in the primary salivary glands of whiteflies. The exploration of highly expressed unigenes suggests that protein processing and secretion are active in the primary salivary glands. Furthermore, we have identified a number of putative secretory proteins that were speculated to play critical roles during whitefly feeding. These analyses provide a valuable resource for future investigations of the functions of salivary gland specific genes and biological processes during whitefly-plant interactions.

## Materials and Methods

### Whitefly Cultures, Primary Salivary Glands Collection and RNA Isolation

Stock cultures of the MED whitefly (mtCO1 GenBank accession no: DQ473394) were maintained on cotton, *Gossypium hirsutum* L. cv. Zhe-Mian 1793, in climate chambers at 27±1°C, a photoperiod of 14 h: 10 h darkness and 70±10% relative humidity. The purity of the cultures was monitored every 3–5 generations using the random amplified polymorphic DNA-polymerase chain reaction (RAPD-PCR) technique with the primer H16 (5′-TCTCAGCTGG-3′) [93]. For RNA extraction, about 120 kidney-shaped primary salivary glands were dissected from the MED adults in PBS (pH7.2) (Invitrogen, USA) on glass slices and total RNA of all glands was isolated using the Absolutely RNA Nanoprep Kit (Agilent, USA) according to the manufacturer's manual.

### Library Construction of the Primary Salivary Glands

The primary gland cDNA library was prepared using a SMARTer™ PCR cDNA Synthesis Kit (Clontech, USA) and a Advantage 2 PCR Kit (Clontech, USA) following the instruction of the kits with slight modification. For first-strand cDNA synthesis, a 3.5 µl aliquot of total RNA (about 80 ng) was mixed with 1 µl of 12 µM 3′ SMART CDS Primer II A. The mixture was incubated at 72 °C for 3 min and then 42 °C for 2 min in a hot-lid thermal cycler. After adding 5.5 µl Master Mix (2 µl 5×First-Strand Buffer, 0.25 µl 100 mM DTT, 1 µl 10 mM dNTP, 1 µl 12 µM SMARTer II A Oligonucleotide, 0.25 µl RNase inhibitor, 1 µl SMARTScribe™ reverse transcriptase), the reaction was incubated at 42 °C for 90 min and then terminated by heating at 70°C for 10 min. The first-strand cDNA product was used for PCR amplification using the following procedures. Two µl of first-strand cDNA combined with reaction reagents (10 µl 10×Advantage 2 PCR Buffer, 2 µl 10 mM 50×dNTP, 4 µl 12 µM 5′ PCR Primer II A, 2 µl 50×Advantage 2 Polymerase and 80 µl deionized water) were subjected to the thermal cycling program: 95 °C for 1 min and variable number of cycles of 95 °C for 15 s, 65 °C for 30 s and 68 °C for 6 min [80]. In order to determine the optimal number of PCR cycles, each 5 µl products of different amplification cycles (15, 18, 21, 24 and 27) of the PCR reaction were electrophoresed. Based on the analysis, 2 µl of first-strand cDNA was amplified in the PCR program of 26 thermal cycles and the reaction was terminated by adding 2 µl 0.5 M EDTA in 100 µl PCR products. After purification of the amplifying cDNA using a QIAquick PCR Purification Kit (Qiagen, Germany), the library for transcriptome sequencing was prepared using Illumina’s kit following manufacturer recommendations.

### Illumina Sequencing, Unigene Annotation

The cDNA library was sequenced in Beijing Genome Institute (Shenzhen, China). The size of the library is approximately 200 bp and both ends of the libraries were sequenced. The raw reads cleaned by removing adaptor sequences, empty reads and low quality sequences (reads with unknown sequences ‘N’), were randomly clipped into 21 bp K-mers for assembly using de Bruijn graph and SOAPdenovo software [94]. Un**igenes** were used for BLAST search and annotation against nr database and Swissprot database with an E-value cut-off of 1.0E^−5^. Functional annotation by GO terms (http://www.geneontology.org) was analyzed by Blast2GO software. Pathway annotation was performed using Blastall software against the KEGG database. The data sets are available at the NCBI Short Read Archive (SRA) with the accession number: SRR316271. The assembled sequences have been deposited in the Transcriptome Shotgun Assembly (TSA) database at NCBI and can be searched using the Gene-ID listed in Table S1.

### Identification of Statistically Enriched Ontologies and Pathways

GO enrichment analysis is an appropriate approach that can recognize the main biological functions of salivary gland genes. In this approach, the hypergeometric test was used to measure significantly enriched GO terms in the salivary gland gene groups in comparison with the whole transcriptome background [45]. The calculating formula is defined as follows: 
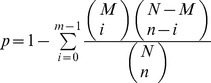
 where N, n respectively indicates the number of whole-body genes and salivary gland genes with GO annotations, and M, m are the number of two sets of genes annotated to a certain GO term (M−m≥0). The GO terms with the *p*-value cut-off of 5.0E^−3^ were deemed to be enriched. In addition, to identify the enriched pathways of the primary salivary glands, the hypergeometric test was used in a similar way to measure the relative coverage of the annotated KEGG orthologous groups of a pathway in the MED whitefly transcriptome background, and the pathways with a *p*-value cut-off of 5.0E^−3^ were determined as enriched [45], [46]. Only the pathways associated with insects are shown in Table 3.

### Secretory Protein Prediction

To translate nucleotide sequences of the unigenes into protein sequences, we first searched all the unigene sequences against protein databases using BLASTx (E-value<1.0E^−5^) in the following order: nr, SwissProt, KEGG and clusters of orthologous groups (COG) database until the sequence had a significant hit. The BLAST results were used to extract coding sequences (CDSs) from unigenes. The CDS of the unigenes that has no significant hit in BLAST search were predicted by ESTScan [95]. Prediction of signal peptides and cleavage sites of the translated unigene sequences was carried out using the SignalP 3.0 Server (http://www.cbs.dtu.dk/services/SignalP-3.0/). Subsequently, we submitted each translated unigene with a signal peptide to the TMHMM Server (http://www.cbs.dtu.dk/services/TMHMM/) for transmembrane domain prediction. The putative protein that has a signal peptide and with no or one transmembrane domain (the transmembrane domain is the predicted signal peptide) would be considered as a potential secreted protein [77].

### Identification of the Homologous Sequences of Aphid Saliva/secretome

All the protein or mRNA sequences that have an association with aphid saliva or secretome were summarized [30], [60], [61], [70], [73], [75], [77]. The predicted protein sequences of the whitefly salivary gland transcriptome were searched using BLASTp and tBLASTn against the protein and mRNA sequences related to aphid saliva or secretome. The unigenes of whitefly salivary glands returning a significant BLAST hit (E-value≤1.0E^−5^) and the matched aphid saliva/secretome sequences are listed in Table S5.

### Analysis of Differential Gene Expression by Statistical Comparison

Gene expression levels can be estimated from Illumina sequencing based on the number of reads for a gene with great accuracy [78], [79]. This approach usually relied on known reference genome for reads mapping. However, the reference information is absent in our transcriptomes, consequently, frameshift and redundancy may affect the mapping of unique reads to genes [81]. To avoid these adverse effects on reads counting, we modified the method according to Chen *et al.* [81] for the measurement of gene expression values. MegaBLAST was used to select orthologous gene pairs with the identity higher than 99% and minimum overlapping region ≥150 bp from the salivary gland and the whole-body transcriptomes. The overlapping regions of the gene pairs were clipped out and the clean reads from the whole-body and salivary gland transcriptomes were mapped to this region, respectively. The number of reads mapped was extracted for comparison. Since read mapping is sensitive to the size of the sequence, we adjusted the raw read count by the total number of reads mapped and the length of the gene by calculating RPKM [78]. Statistical comparison between the salivary gland and the whole-body transcriptomes was performed with a custom written script using the algorithm described by Audic *et al.* [96]. Here, we used FDR<1.0E^−3^ and the absolute value of log_2_ratio≥1 as the threshold to judge the significance of the gene expression difference. All the data of the MED whitefly whole-body transcriptome used in this analysis were obtained from NCBI [37]. These data were originally produced by our laboratory, and the insects used for the whole-body transcriptome sequencing were reared under the same host plants and climatic conditions as those for rearing insects for this study (see above).

### qPCR Analysis

To confirm the results of the RPKM comparison, the expression profiles of 20 selected genes of the primary salivary glands and the adult whole body (minus primary salivary glands) were measured using qPCR. The RNA samples of the primary salivary glands and the whole body were extracted and then, both samples were used for first-strand cDNA synthesis with a PrimeScript RT reagent Kit (Takara, Japan). Amplification of those cDNA samples was carried out in Bio-Rad CFX96TM Real-Time System (Bio-Rad, USA) using SYBR Premix Ex Taq TM II (Takara, Japan). The cycling parameters were 95°C for 30 seconds followed by 40 cycles of 95°C for 5 s and 60°C for 35 s. For each gene, three replicates were analyzed and the average threshold cycle (C*_t_*) was calculated. Finally, the relative expression level was calculated using the 2^−ΔΔ*Ct*^ method. The TAF10 RNA polymerase II, TATA box binding protein (TBP)-associated factor gene (tbp-af) was chosen as the endogenous reference gene in qPCR analysis for the following two reasons. First, it has been used in qPCR analyses of samples from different tissues [97]. Second, RPKM analysis has shown that the tbp-af gene (BT_Q_SG_ZJU_Unigene13122) was expressed at the same level in the primary salivary glands (RPKM: 2217.95) and the whole body (RPKM: 2371.84). All the primers for qPCR analysis are shown in Table S8.

## Supporting Information

Figure S1 **Size distribution of unigenes from the primary salivary gland transcriptome of the MED whitefly.**
(EPS)

Table S1 **Top BLAST hits from NCBI nr database.** BLAST results against the NCBI nr database for all the unigenes with a cut-off E-value <1.0E^−5^ are shown.
(XLS)

Table S2 **Statistically enriched Gene Ontology terms in the “Biological Process=" category.** SG genes: the number of primary salivary gland genes that belong to each GO. WB genes: the total number of whole-body genes that belong to each GO.
(DOC)

Table S3 **Statistically enriched Gene Ontology terms in the “Cellular Component=" category.** SG genes: the number of primary salivary gland genes that belong to each GO. WB genes: the total number of whole-body genes that belong to each GO.
(DOC)

Table S4 **Genes with a putative signal peptide.** Based on the coding sequence of translated unigenes, the presence of a signal peptide was predicted using SignalP. v. 3.0 in addition to the calculation of cleavage position, resulting in 356 proteins with putative secretion signals. These protein sequences were assigned to transmembrane domain (TMH) prediction with the TMHMM Server v. 2.0. The annotations were acquired through searching unigenes against nr, Swissprot and COG database.
(XLS)

Table S5 **Unigenes of the whitefly primary salivary glands with similarity to the sequences related to aphid secretory proteins.** Predicted protein sequences of the whitefly primary salivary glands were BLAST searched against the set of protein or mRNA sequences associated with aphid saliva or secretome. The unigenes that are homologous to the sequences of the proteins of aphid saliva or secretome (E-value ≤1.0E^−5^) are shown. The unigenes marked in red are also predicted to be secretory proteins.
(XLS)

Table S6 **Differentially expressed genes in statistical analysis of RPKM value.** The gene pairs having ≥150 overlapping region and ≥99% identity are shown. RPKM, FDR-value, fold change (log_2_ ratio) of gene expression and best hits against nr and Swissprot database (E-value <1.0E^−5^) for all the gene pairs are also listed in this table.
(XLS)

Table S7 **Gene expression data verified by qPCR.** Twenty unigenes were selected for validation of expression level using qPCR.
(XLS)

Table S8 **Primers for qPCR.** The primers of the 20 selected unigenes and the reference gene (BT_Q_SG_ZJU_Unigene13122) used for validation of expression level were listed.
(XLS)
