# Supplementary material for: Transcriptomic Analysis of the Salivary Glands of an Invasive Whitefly
Source: PLoS One. 2012 Jun 20;7(6):e39303. doi: 10.1371/journal.pone.0039303 (PMC3379992; doi:10.1371/journal.pone.0039303)
Supplement: Table S2 — Statistically enriched Gene Ontology terms in the “Biological Process=" category. SG genes: the number of primary salivary gland genes that belong to each GO. WB genes: the total number of whole-body genes that belong to each GO. (DOC) [file pone.0039303.s003.doc]

**Table S2: Statistically enriched Gene Ontology terms in the “Biological Process” category**

| GO ID | SG Genes | WB Genes | *p*-value | GO Ontology or Category |
| --- | --- | --- | --- | --- |
|
| 0050896 | 47 | 79 | 8.00E-14 | Response to stimulus |
| 0007165 | 32 | 56 | 7.23E-05 | Signal transduction |
| 0051179 | 154 | 351 | 8.00E-14 | Localization |
| 0008104 | 28 | 50 | 3.13E-06 | Protein localization |
| 0034613 | 18 | 23 | 2.99E-05 | Cellular protein localization |
| 0051641 | 26 | 37 | 1.40E-09 | Cellular localization |
| 0051234 | 147 | 348 | 3.04E-09 | Establishment of localization |
| 0045184 | 28 | 47 | 4.48E-05 | Establishment of protein localization |
| 0051649 | 26 | 37 | 8.74E-07 | Establishment of localization in cell |
| 0006810 | 147 | 348 | 2.57E-06 | Transport |
| 0015031 | 28 | 47 | 3.98E-05 | Protein transport |
| 0046907 | 20 | 30 | 3.40E-05 | Intracellular transport |
| 0006886 | 18 | 23 | 5.71E-04 | Intracellular protein transport |
| 0006605 | 5 | 5 | 4.27E-03 | Protein targeting |
| 0044085 | 27 | 45 | 2.33E-11 | Cellular component biogenesis |
| 0034622 | 13 | 19 | 2.07E-04 | Cellular macromolecular complex assembly |
| 0043623 | 8 | 10 | 2.56E-03 | Cellular protein complex assembly |
| 0008152 |  |  |  | Metabolic process |
| 0005975 | 49 | 102 | 5.59E-06 | Carbohydrate metabolic process |
| 0006066 | 25 | 47 | 5.59E-06 | Alcohol metabolic process |
| 0046164 | 14 | 20 | 2.07E-04 | Alcohol catabolic process |
| 0009056 | 48 | 76 | 4.01E-11 | Catabolic process |
| 0009057 | 19 | 19 | 1.01E-07 | Macromolecule catabolic process |
| 0016052 | 16 | 24 | 2.63E-06 | Carbohydrate catabolic process |
| 0044275 | 14 | 21 | 1.31E-03 | Cellular carbohydrate catabolic process |
| 0044237 | 388 | 1053 | 5.28E-09 | Cellular metabolic process |
| 0006139 | 175 | 368 | 1.87E-07 | Nucleobase, nucleoside, nucleotide and nucleic acid metabolic process |
| 0044260 | 255 | 626 | 2.24E-06 | Cellular macromolecule metabolic process |
| 0006259 | 39 | 75 | 1.23E-03 | DNA metabolic process |
| 0006281 | 14 | 23 | 2.58E-03 | DNA repair |
| 0016070 | 96 | 163 | 3.29E-04 | RNA metabolic process |
| 0032774 | 54 | 82 | 8.12E-06 | RNA biosynthetic process |
| 0006396 | 23 | 34 | 1.08E-03 | RNA processing |
| 0034660 | 29 | 54 | 2.53E-03 | ncRNA metabolic process |
| 0006351 | 53 | 82 | 3.29E-04 | Transcription, DNA-dependent |
| 0044248 | 41 | 52 | 1.25E-08 | Cellular catabolic process |
| 0051186 | 30 | 34 | 3.86E-08 | Cofactor metabolic process |
| 0006732 | 21 | 24 | 8.12E-06 | Coenzyme metabolic process |
| 0006730 | 10 | 10 | 1.08E-06 | One-carbon metabolic process |
| 0044262 | 31 | 49 | 1.52E-06 | Cellular carbohydrate metabolic process |
| 0005996 | 22 | 25 | 3.58E-04 | Monosaccharide metabolic process |
| 0019318 | 21 | 24 | 3.61E-04 | Hexose metabolic process |
| 0006096 | 12 | 16 | 2.07E-04 | Glycolysis |
| 0006790 | 9 | 10 | 2.32E-06 | Sulfur metabolic process |
| 0006457 | 19 | 30 | 1.23E-03 | Protein folding |
| 0065007 |  |  |  | Biological regulation |
| 0050789 | 96 | 192 | 8.53E-11 | Regulation of biological process |
| 0009889 | 55 | 98 | 2.29E-05 | Regulation of biosynthetic process |
| 0050794 | 94 | 169 | 1.01E-07 | Regulation of cellular process |
| 0031323 | 58 | 101 | 2.01E-05 | Regulation of cellular metabolic process |
| 0031326 | 55 | 98 | 5.45E-04 | Regulation of cellular biosynthetic process |
| 0019219 | 55 | 96 | 5.20E-04 | Regulation of nucleobase, nucleoside, nucleotide and nucleic acid metabolic process |
| 0019222 | 60 | 105 | 7.50E-07 | Regulation of metabolic process |
| 0060255 | 59 | 104 | 2.01E-05 | Regulation of macromolecule metabolic process |
| 0010556 | 55 | 98 | 5.45E-04 | Regulation of macromolecule biosynthetic process |
| 0051252 | 52 | 66 | 2.59E-03 | Regulation of RNA metabolic process |
| 0010468 | 57 | 98 | 4.64E-04 | Regulation of gene expression |
| 0006355 | 52 | 66 | 4.85E-03 | Regulation of transcription, DNA-dependent |
| 0042592 | 12 | 19 | 5.59E-06 | Homeostatic process |
| 0019725 | 12 | 18 | 3.26E-09 | Cellular homeostasis |
