# Supplementary material for: Transcriptomic Analysis of the Salivary Glands of an Invasive Whitefly
Source: PLoS One. 2012 Jun 20;7(6):e39303. doi: 10.1371/journal.pone.0039303 (PMC3379992; doi:10.1371/journal.pone.0039303)
Supplement: Table S3 — Statistically enriched Gene Ontology terms in the “Cellular Component=" category. SG genes: the number of primary salivary gland genes that belong to each GO. WB genes: the total number of whole-body genes that belong to each GO. (DOC) [file pone.0039303.s004.doc]

**Table S3: Statistically enriched Gene Ontology terms in the “Cellular Component” category**

| GO ID | SG Genes | WB Genes | *p*-value | GO Ontology |
| --- | --- | --- | --- | --- |
| 0005886 | 54 | 113 | 7.96E-04 | Plasma membrane |
